# Excess selenium intake is associated with microalbuminuria in female but not in male among adults with obesity: Results from NHANES 2009–2018

**DOI:** 10.3389/fnut.2023.1043395

**Published:** 2023-01-25

**Authors:** Jia-wei Zhang, Yi Lin, Yue-min Liu, Min-min Wang, Jian-guang Gong, Xiao-gang Shen, Quan-quan Shen, Bo Lin, Wei-er Su, Yuan-cheng Gao, Chen-yi Yuan, Zhi-hui Pan, Bin Zhu

**Affiliations:** ^1^Department of Nephrology, Zhejiang Provincial People’s Hospital, Affiliated People’s Hospital, Hangzhou Medical College, Hangzhou, China; ^2^Department of Nephrology, Hangzhou Hospital of Traditional Chinese Medicine (Guangxing Hospital), Affiliated to Zhejiang Chinese Medical University, Hangzhou, China; ^3^The Third College of Clinical Medicine, Zhejiang Chinese Medical University, Hangzhou, China

**Keywords:** dietary selenium, microalbuminuria, chronic kidney disease (CKD), National Health and Nutrition Examination Survey (NHANES), epidemiology

## Abstract

**Introduction:**

Selenium is a critical trace element with antioxidant activities that has been related to the preservation of kidney function. Few studies, however, have looked at the effects of excess selenium on kidneys. The purpose of the present study was performed to investigate the relationship between dietary selenium intake and the prevalence of microalbuminuria in American adults with obesity.

**Methods:**

A total of 8,547 participants with obesity in the National Health and Nutrition Examination Survey (NHANES) with the age of 19 years or older were included in the present study. Multivariable regression and subgroup analyses were performed to examine the association between dietary selenium and microalbuminuria in the two genders, separately. A selenium intake above the median was defined as high selenium intake.

**Results:**

Dietary selenium intake was significantly higher in men compared to women (139.49 μg/day vs. 101.06 μg/day; *P* < 0.0001). Among female participants, the prevalence of microalbuminuria was significantly higher in participants with a high selenium intake compared with those without a high selenium intake (13.82 vs. 9.96%; *P* = 0.008), whereas this difference did not exist in male participants (10.79 vs. 11.97%; *P* = 0.40). Dietary selenium is not significantly correlated with microalbuminuria (*P* = 0.68) in the male population, whereas each 1 μg/day of increase in selenium consumption was independently associated with a 6h higher risk of microalbuminuria (OR = 1.006; 95% CI, 1.001–1.011, *P* = 0.01) in females.

**Conclusion:**

According to our research, excessive selenium consumption is positively correlated with microalbuminuria in females with obesity, but not in males with obesity.

## 1. Introduction

Obesity is a growing global public health concern, and it is spreading dramatically along with economic development. It is widely acknowledged as a chronic metabolic disorder associated with hypertension, diabetes, chronic kidney disease (CKD), and cardiovascular disease (CVD) (1–3). Estimates from the World Health Organization’s (WHO) showed that obesity claims the lives of at least 2.8 million people annually, and poses a serious threat to both the economy and public health (4). Microalbuminuria is a hallmark of nephropathy, which is defined as an increase in the urinary albumin-to-creatinine ratio (UACR) above 30 mg/g (5). As a biomarker of glomerular filtration barrier damage, UACR has been integrated into the KDIGO criteria for the diagnosis of CKD (6). Microalbuminuria is directly related to obesity (1, 7). Recently, it has been found that more than 20% of people with obesity developed microalbuminuria in New York (8).

Micronutrients are regarded as a crucial part of nutritional therapies (9). Selenium, an essential trace element identified in 25 different types of selenoprotein in human, is extremely important for health (10, 11). Abnormal selenoprotein level is associated with a variety of diseases, such as type 2 diabetes, neuronal degeneration, CKD, CVD, and cancer (12–14). Previous studies suggest that selenium supplementation might improve the prognosis of CKD and CVD patients with selenium deficiency (13, 15).

Selenium has been demonstrated to play an important role in the metabolism for carbohydrates and lipids that was involved in the regulation of body weight and energy metabolism (16, 17). Selenium deficiency can cause pancreatic apoptosis, reduction of serum insulin, glucagon levels, and decreased antioxidant enzyme activity, as well as an increase of free radicals (18). A population-based study has found that low whole-blood glutathione peroxidase (GPx) activity is associated with higher levels of both general and central adiposity (19). It was also demonstrated that decreased serum selenium level was related to obesity in the females (20).

Previous studies showed that either insufficient or excessive selenium intake has been associated with some diseases (21). There was also a U-shaped relationship between selenium levels and the severity of diseases such as diabetes and cardiometabolic diseases. These indicated that selenium supplementation should be limited in a specific range (22, 23). The recommended dietary allowance (RDA) for selenium is 55 μg/day in the United States (24). The selenium intake of up to 400 μg per day are generally not harmful (25). However, it is still controversial whether the higher the selenium intake the more benefits for human at the non-toxic doses.

Some studies suggested that selenium effects were sex-specific. Combs et.al indicated despite higher selenium intakes in men compared with women, the selenium levels in serum, whole blood, and toenail are similar between the two genders (26). However, Rodriguez et al. found that the selenium levels in the plasma and urine in the females were higher than those in the males (27). Male mice receiving diets with a high selenium level had a 31% lower level of adiposity than that in female mice (28). Cardoso et al. found a correlation between blood selenium levels and cognitive function in men but not in women (29). Serum selenium levels were associated with cardiovascular mortality only in female but not in male persons (30). Furthermore, orchiectomy has been demonstrated to alleviate sex-specific differences in the expression of selenoproteins (31). These suggest that the sex hormones may be involved in the sex-specific biological functions of selenium.

So far, the relationship between the high dietary selenium intake at non-toxic doses and microalbuminuria has not been clarified, especially in the people with obesity, who were at risk of CKD.

The aim of the present study is to investigate the association between dietary selenium consumption and microalbuminuria in US adults with obesity.

## 2. Materials and methods

### 2.1. Population

**FIGURE 1:**
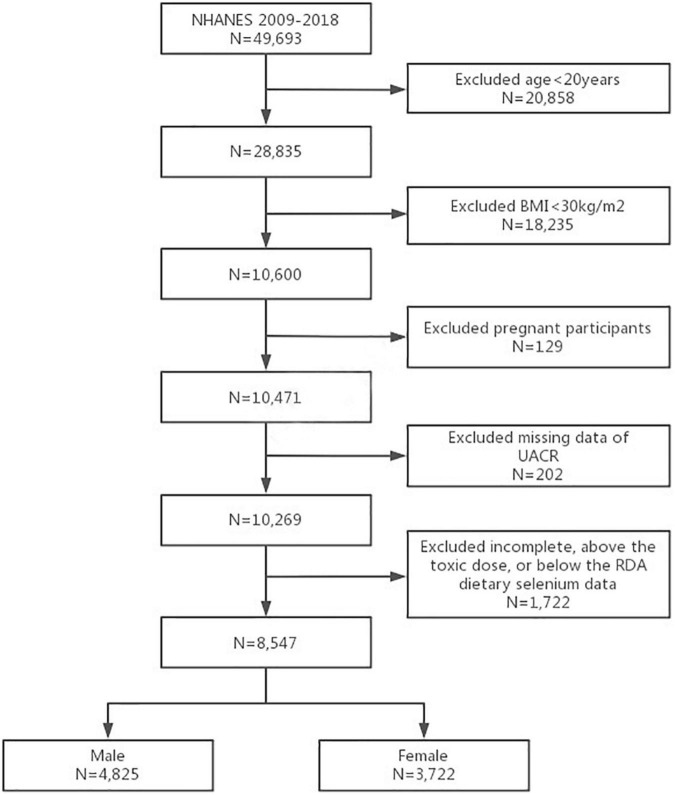
Flowchart of the sample selection from NHANES 2009–2018.

This study was conducted using data from the National Health and Nutrition Examination Survey (NHANES) database. The Centers for Disease Control and Prevention (CDC)’s NHANES is a freely accessible database with information from a continuously collected, nationally representative cross-sectional survey of non-institutionalized civilian residents of the United States using a complex, stratified, multistage probability cluster sampling design. Interview, physical, and laboratory data are included in the dataset, which can be downloaded from the NHANES website.^1^ All NHANES participants over the age of 16 had signed a written informed consent form in person. As the NHANES study protocols had already been approved by the NCHS Research Ethics Review Board, no additional ethical clearance was required for the present study (32). A total of 49,693 NHANES participants from 2009 to 2018 was screened. We screened those who were 20 years old or over and with obesity [defined as the body mass index (BMI) greater than 30kg/m^2^ (4)] (*n* = 10,600) from all participants. We further excluded those with pregnancy (*n* = 129) or missing information of urinary albumin and creatinine assessments (*n* = 202). Participants with incomplete selenium data, or with selenium intake above the toxic dose (>400 μg/day) (24) were also excluded (*n* = 1,722) (25) from the analyses. Finally, a total of 8,547 adults (4,825 female, 3,722 male) were included in the present study (Figure 1).

### 2.2. Urinary albumin-to-creatinine ratio and microalbuminuria

Participants in the NHANES had their urine samples taken at a standardized mobile examination center (MEC). Urinary albumin and creatinine levels were measured using a solid-phase fluorescent immunoassay and modified Jaffe kinetic method. UACR was obtained by dividing the urinary albumin concentration in milligrams by the urinary creatinine concentration in grams. Microalbuminuria was defined as UACR over 30 mg/g (6).

### 2.3. Dietary selenium intake

Dietary data were gathered by the interviewers received intensive training programs. Two interviews of 24-h dietary recall were adopted for the determination of the total dietary selenium intake. All participants underwent in-person interviews for the initial 24-h dietary recall interview. Then, 3–10 days later, a portion of the adult participants took part in a second, telephone-based 24-h dietary recall interview. In the present study, only the participants with two dietary recall statuses available were included.

### 2.4. Covariates

The included covariates were age, race/ethnicity, educational background, income level, total energy intake, smoking, drinking, hypertension, diabetes, CVD, waist circumference (WC; cm), and serum creatinine (SCr; mg/dL). Race/ethnicity was categorized as non-Hispanic White, non-Hispanic Black, Mexican-American, and other races. Smoking levels was categorized as non-smoker (less than 100 cigarettes in life), former or occasional smoker (a total of more than 100 cigarettes in life, but not as a daily habit), or daily smoker. Drinking status was categorized as non-drinker (less than 12 alcoholic drinks per year), former or light drinker (more than 12 alcoholic drinks per year but no more than 1 alcoholic drink per day), or heavy drinker (more than 1 alcoholic drink per day). Educational backgrounds were categorized as less than high school, high school graduation/general educational development (GED), and College graduate or above. Family income-to-poverty ratio (FIPR) was used to categorize income levels into three groups: poor (FIPR < 1.3), middle (FIPR 1.3–3.5), and wealthy (FIPR > 3.5). Hypertension status was classified as normal (<140/90 mmHg), grade 1 (140/90–159/99 mmHg), grade 2 (160/100–179/109 mmHg), and grade 3 (≥180/110 mmHg) based on self-reported hypertension and/or systolic and diastolic blood pressure measurements in MEC (33). Participants were deemed to have diabetes when they self-reported diabetes, or were with HbA1c level over 6.5% (34). CVD status was determined according to self-reported medical issues of heart failure, coronary heart disease, angina/angina pectoris, heart attack, or stroke.

### 2.5. Statistical analyses

All statistical analyses were performed using SAS OnDemand for Academics (SAS Institute Inc., Cary, NC, USA) and R software (version 4.0.3). Given that dietary selenium intake, as well as the effects of selenium were different between the two genders (29, 35), we examined the association between dietary selenium and microalbuminuria in the male and female participants, separately. Continuous variables with normal distribution are presented as means ± standard errors (SEs), and those with abnormal distribution as medians and interquartile ranges. Categorical variables are presented as proportions. The dietary selenium intake levels were log10-transformed to satisfy the assumption of normality. Differences in continuous variables were assessed using weighted *t*-tests. Weighted chi-squared tests were used to analyze differences in categorical variables. Logistic regression analysis was performed to investigate the relationship between the total intake of dietary selenium and the prevalence of microalbuminuria by calculation of the odds ratio (OR) and 95% confidence interval (CI). Three models were developed: Model 1 was not adjusted any covariates; Model 2 was adjusted for age, race/ethnicity, educational background, income level, and total energy intake; Model 3 was adjusted for covariates in model 2 plus smoking, drinking, WC, SCr, hypertension, diabetes, and CVD status. Subgroup analyses examined the relationships between dietary selenium intake and the prevalence of microalbuminuria, stratified by covariates of age, hypertension status, diabetes mellitus status, and SCr level. *P*-values for interaction were derived from the multivariable logistic regression model with an interaction term.

## 3. Results

### 3.1. Baseline characteristics of the study population

The present study included a total of 8,547 adult participants (4,825 female, 3,722 male). Consistent with previous reports (26), there were significant differences in dietary selenium intake between men and women in the present study (*P* < 0.0001). The daily intake of selenium in all participants was 117.49 (85.56, 158.47) μg. The selenium intake in the male participants was 139.49 (104.53, 182.90) μg/d, which was significantly higher than 101.06 (73.86, 133.05) μg/d in the female participants (Table 1). According to the median values (139.49 μg/day for male and 101.06 μg/day for female) of selenium intake in male and female participants, we divided male and female participants into low/high selenium intake groups, separately. The demographic and clinical characteristics of the participants according to the sex and selenium intake group were shown in Table 2. The prevalence of microalbuminuria was significantly higher in the high-selenium-intake group than that in the low-selenium-intake group in the female participants (*P* = 0. 01). However, there is no significant difference between groups in the male participants (*P* = 0. 40).

**TABLE 1:** Dietary selenium intake among US adults (>19 years) in NHANES 2009–2018.

	Total sample population (*n* = 8,547)	Male (*n* = 3,722)	Female (*n* = 4,825)	P*
Dietary selenium intake (μg/d)	117.49 (85.56, 158.47)	139.49 (104.53, 182.90)	101.06 (73.86, 133.05)	<0.0001

*The *P*-value is for the difference between the male and the female participants.

**TABLE 2:** Characteristics of the sample population from NHANES 2009–2018.

Characteristic	Male			Female		
	Dietary Se intake ≤139.49^a^μg/day (*n* = 2,017)	Dietary Se intake >139.49^a^μg/day (*n* = 1,705)	*P*	Dietary Se intake ≤101.06^a^μg/day (*n* = 2,531)	Dietary Se intake >101.06^a^μg/day (*n* = 2,294)	*P*
Age (years)	48.49 ± 0.56	47.23 ± 0.52	0.10	49.76 ± 0.52	49.69 ± 0.51	0.93
Race/ethnicity (%)			0.05			0.63
Mexican American	10.40	11.51		10.44	10.10	
Non-Hispanic White	64.98	66.76		59.51	61.93	
Non-Hispanic Black	12.78	9.30		18.37	16.81	
Others^b^	11.84	12.44		11.67	11.15	
Education background (%)			<0.0001			<0.0001
Below high school	16.87	10.69		19.34	12.66	
High school graduate or some college^c^	59.85	60.77		62.11	59.87	
College graduate or above	23.28	28.54		18.55	27.48	
Income level (%)			0.002			<0.0001
Poor	21.01	15.06		33.57	23.06	
Middle	36.57	35.83		38.07	37.01	
Rich	42.42	49.11		28.36	39.94	
Drinking status (%)			0.42			0.02
Never	12.30	10.75		32.20	26.47	
Former or light	30.31	28.28		32.28	34.63	
Heavy	57.39	60.97		35.52	38.90	
Smoking status (%)			0.15			0.001
Never	48.48	50.81		60.04	63.35	
Not daily	38.20	39.04		24.27	26.49	
Daily	13.32	10.15		15.69	10.17	
Hypertension (%)			0.85			0.41
Non-hypertension	48.51	48.31		51.21	48.83	
Grade 1	46.93	47.77		44.54	46.86	
Grade 2	3.54	2.80		3.36	3.78	
Grade 3	1.02	1.12		0.88	0.54	
MAP (mmHg)	92.05 ± 0.32	91.85 ± 0.46	0.69	87.93 ± 0.32	89.39 ± 0.39	0.007
Diabetes (%)	21.57	18.61	0.19	21.02	18.16	0.11
HbA1c (%)	5.91 ± 0.04	5.88 ± 0.05	0.70	5.85 ± 0.03	5.90 ± 0.04	0.31
CVD (%)	13.21	10.35	0.07	11.77	9.32	0.09
WC^d^ (cm)	117.39 ± 0.43	116.77 ± 0.50	0.29	112.41 ± 0.44	113.56 ± 0.44	0.07
SCr (mg/dL)	1.01 ± 0.01	1.00 ± 0.01	0.33	0.80 ± 0.01	0.78 ± 0.01	0.04
Total energy intake (kcal)	1971.76 ± 20.89	2813.27 ± 29.67	<0.0001	1472.36 ± 13.79	2087.00 ± 16.07	<0.0001
UACR (mg/g)	6.25 (3.95, 12.06)	5.87 (3.86, 11.35)	0.33	7.91 (5.18, 13.33)	7.67 (5.05, 15.21)	0.06
Microalbuminuria (%)	11.97	10.79	0.40	9.96	13.82	0.008

^a^Sex-specific median dietary selenium intake; ^b^Other hispanics and other races including multi-racial participants; ^c^General educational development; ^d^Waist circumference.

### 3.2. Dietary selenium intake and the incidence of microalbuminuria

**FIGURE 2:**
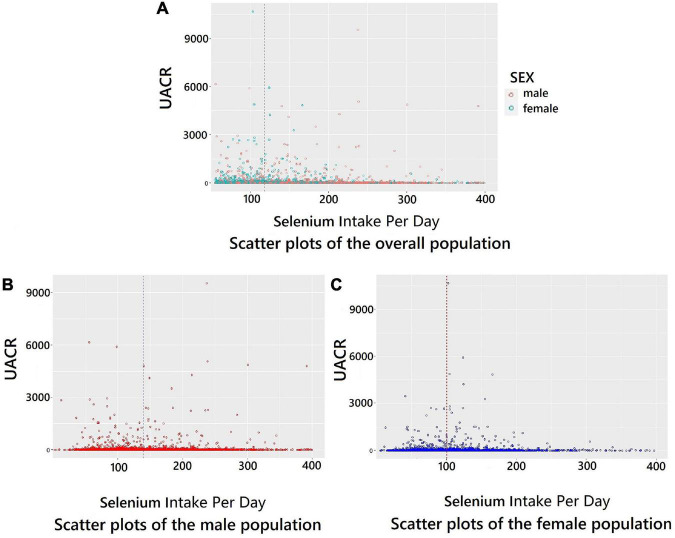
Scatter plots for dietary selenium intake per day and UACR. **(A)** Scatter plots of the overall population. **(B)** Scatter plots of the male population. **(C)** Scatter plots of the female population. The vertical dashed line indicates the median value of dietary selenium intake.

Scatterplots of dietary selenium intake vs. UACR for the overall population, men, and women were presented in Figure 2. We further performed logistic regression analyses to investigate the relationship between the incidence of microalbuminuria and dietary selenium intake levels. As shown in Table 3, the increase of daily dietary selenium intake is significantly associated with a higher risk of microalbuminuria in female participants in both the univariate and multivariate models adjusted for covariates of age, race/ethnicity, education level, income level, total energy intake, smoking, alcohol consumption, WC, SCr, hypertension status, diabetes status, and CVD status (OR = 1.005; 95% CI: 1.001–1.009, *P* = 0.02 in the univariate model and OR = 1.006; 95% CI: 1.001–1.011 *P* = 0.01 in the multivariate model by 1 μg of increase in daily dietary selenium). However, there was no significant association between dietary selenium and microalbuminuria in the male participants.

**TABLE 3:** Association between dietary selenium and microalbuminuria.

	Model 1^a^		Model 2^b^		Model 3^c^	
	OR^d^ (95% CI^e^)^f^	*P*	OR (95% CI)^f^	*P*	OR (95% CI)^f^	*P*
Male	0.999 (0.996, 1.001)	0.23	1.000 (0.997, 1.004)	0.80	1.001 (0.997, 1.004)	0.68
Female	1.005 (1.001, 1.009)	0.02	1.006 (1.001, 1.010)	0.01	1.006 (1.001, 1.011)	0.01

^a^Model 1: No covariates were adjusted.

^b^Model 2: Adjusted for age, and race/ethnicity, education background, income level, total energy intake.

^c^Model 3: Adjusted for age, race/ethnicity, education background, income level, total energy intake, smoking status, drinking status, WC, SCr, hypertension status, diabetes status, CVD status.

^d^OR, odds ratio.

^e^95% CI, 95% confidence interval.

^f^OR (95% CI) according to selenium intake (μg/day).

### 3.3. Subgroup analyses

**FIGURE 3:**
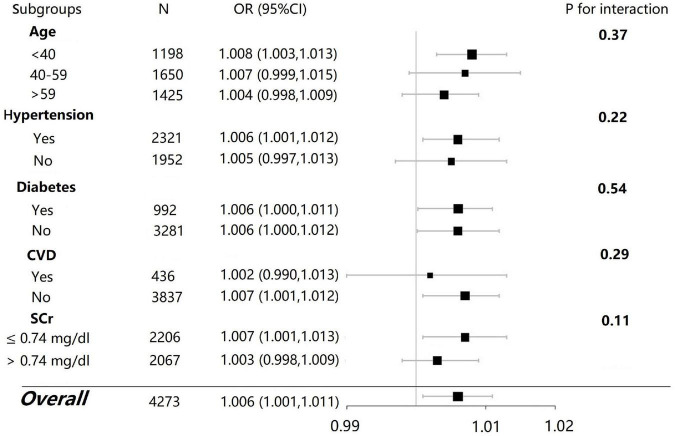
Subgroup analysis for the association between dietary selenium intake and microalbuminuria in female participants.

Subgroup analyses were performed by the stratified factors such as age (<40, 40–59, and > 59), hypertension (yes/no), diabetes (yes/no), and SCr levels (separated at the median cutoff point). The associations between dietary selenium intake and microalbuminuria were inconsistent between subgroups of female participants (Figure 3). There were more significant associations between dietary selenium intake and microalbuminuria in those who were younger than 40 years old, with hypertension, without diabetes, without cardiovascular disease, and with an SCr level lower than 0.74 mg/dL (*P* < 0.05) as compared with the other subgroup, respectively. However, the interaction effects were not significant between the subgroups. There were no statistically significant findings in the subgroup analyses in the male participants (data not shown).

## 4. Discussion

Using a population-based database of US adults, the present study firstly indicated that excessive selenium consumption was significantly associated with a higher risk of developing microalbuminuria in females with obesity but not in males with obesity.

Selenium supplementation can improve the long-term outcome of CKD patients with selenium deficiency (36). However, a high intake of selenium may injury kidney (37). Serum creatinine and blood urea nitrogen levels were both increased when mice were exposed to diet with excess selenium (38). A previous study (39) found that excess selenium is transformed into selenosugars in liver, and then transported to kidneys to be excreted in urine finally. The excretion of selenium relies on S-adenosylmethionine (SAM)-dependent methylation. In the individuals with obesity, high fatty acid oxidation can attenuate the activity of methionine adenosyltransferase to suppress the transmethylation process, leading to a decreased conversion of selenite and its intermediate metabolites for excretion. Then selenium levels were increased (40). High selenium levels can cause endothelial cell dysfunction due to ER stress and lower NO bioavailability, by the concomitant release of ROS and reduced NO production (41). Glomerular endothelial cells, the essential part of glomerular filtration barrier, may be injured by high selenium levels leading to albuminuria (42). In addition, high selenium levels may increase the risk of kidney ischemia/reperfusion injury. The mRNA levels of kidney thioredoxin reductase, one of the essential multifunctional antioxidant enzymes in kidney, were significant decreased in rats injected intraperitoneally with sodium selenite (43). Thioredoxin system with thioredoxin reductase, as one of the critical components of redox balance system, is important in protecting organs from ischemia/reperfusion damage (44). When the kidney is injured by ischemia and reperfusion, thioredoxin retains in the areas most vulnerable to ischemia-reperfusion for protection. Reperfusion injury is attenuated in kidney in the transgenic mice overexpressing thioredoxin (45). Mice with ischemia/reperfusion develop podocyte damage and extensive foot processes effacement, leading to microalbuminuria (46). Kidneys are susceptible to oxidative stress and ischemia/reperfusion injury due to inhibition of thioredoxin reductase by high selenium levels. Furthermore, in previous study, excess Se intake increased the expression of GPx1, GPx3, GPx4, and SelW in the kidney. The increased selenoproteins levels may lead to an imbalance of oxidative stress to injury kidney (38).

It has been demonstrated that selenium was associated with energy metabolism (16, 17). Selenium enhances GPx1 production to excessively remove hydrogen peroxide, which is one of the key messengers in insulin signaling pathway. Shortage of hydrogen peroxide leads to the disruption of insulin homeostasis (47, 48). Therefore, consumption of appropriate amounts of selenium is important to maintain the stability of body weight and energy metabolism. People with obesity have higher levels of inflammation and chronic oxidative stress with increased reactive oxygen species (ROS) (49), which are closely related to metabolic disease and insulin resistance. Selenium, a high potential antioxidant, can reduce ROS levels, and suppress chronic inflammation (10). Appropriate selenium consumption has become a major focus in nutritional therapy in individuals with obesity. A higher intake of selenium in non-toxic doses for human health is controversial (24).

A number of studies indicated that excessive selenium intake may cause some diseases in human. Excess selenium intake impairs insulin-stimulated signaling to cause glucose tolerance, hyperinsulinemia (50, 51), and hypertension (52). Impaired insulin signaling associated hyperinsulinemia can lead to visceral fat accumulation (53, 54), which is associated with several metabolic diseases and renal filtration barriers injury (3, 55). This may be exacerbated in the population with obesity (56–59). Moreover, we observed that female participants with a high dietary selenium intake had higher levels of WC (*P* = 0.07) and MAP (*P* = 0.007) than those with low dietary selenium intake. This may be partly explained by insulin resistance related hypertension and central obesity in women (54, 59), and the risk of microalbuminuria. People with obesity are at high level of oxidative stress (49) that can be aggravated in high selenium status. Mice with excess selenium presented with a higher level of oxidized red blood cells, and have the worse atherogenic index (60). These suggest that excessive selenium may reduce antioxidant effects in mammals.

In the present study, microalbuminuria was associated with dietary selenium intake in female participants, but not in male participants. This sex-specific effect of selenium for human health has also been demonstrated in several previous studies (29, 30). It was suggested that the association between higher whole blood selenium levels and better cognitive function was only significant in men, whereas the association between higher serum selenium level and lower CVD mortality was only significant in women. Androgens can stimulate the expression of some selenoproteins, such as SELENOP and SePP (61). Then these selenoproteins were expressed higher in the kidney of male mice as compared with female mice, resulting in increased antioxidant activity in the male mice (31). The increased expression of SELENOP and SePP can maintain antioxidant activity in organs to inhibit inflammation. Moreover, a previous study indicated that adult men needed 80 micrograms selenium per day, whereas women needed only 57 mg selenium per day for keeping selenium balance (62). These suggest that selenium may be easily accumulated in women on a high selenium diet, indicating women may be more susceptible to selenium intake associated adverse effects (23, 63).

The subgroup analyses indicated that there was no significant heterogeneity across the prespecified subgroups. In the present study, a high dietary selenium intake was solidly associated with high prevalence of microalbuminuria in younger women, but not in older women. It has been demonstrated that serum selenium levels tend to decline with aging so that the older women require more selenium supplementation (64, 65). This indicates that the recommended selenium intake should vary depending on age. Furthermore, we revealed that a high selenium intake was significantly associated with microalbuminuria only in the hypertensive subgroup. High serum selenium concentrations was demonstrated to be associated with a higher prevalence of hypertension in United States (66). Then, high selenium diet may further increase blood pressure, thus cause microalbuminuria by injuring the glomerular filtration barrier (67). There is insignificant association between excess selenium intake and microalbuminuria in the CVD subgroup. We assume that it may be attributed to the reasons as follows: Patients with CVD tend to be older and have lower serum selenium levels compared with the younger population, and the CVD patients require more selenium intake (64, 65). Also, the insignificance in the CVD subgroup may be induced by the shortage of statistical power by the limited sample size and few microalbuminuria events.

The present study has several strengths. We included a large sample size in the nationally representative database. We evaluate a potential sex-specific response to excess selenium intakes by analyzing in the two genders separately. However, there are some limitations. First, Dietary selenium intake was collected *via* food-frequency questionnaires (FFQs), recall bias, and measurement bias could not be eliminated. Since this is a cross-sectional study, we were unable to establish causality between dietary selenium and microalbuminuria. Although we adjusted for a range of potential confounders, residual confounding may exist. Another limitation of the present study is the lack of measurement of selenium biological biomarkers (such as serum selenium levels, nail selenium concentrations).

There is no intention to reduce selenium intake in the female population with obesity, which would deprive them of the numerous health benefits of supplementation with selenium. The association between excess selenium intake and microalbuminuria of the present study suggests that the range of selenium intake in female with obesity individuals should be carefully limited in an appropriate range. We look forward to further prospective studies on issue of appropriate selenium intake in individuals with obesity to clarify the causal relationship between excessive selenium intake and microalbuminuria.

## 5. Conclusion

High dietary selenium levels were associated microalbuminuria in females but not in males among people with obesity. Further large-scale prospective studies are still needed to validate this finding.

## Data availability statement

The original contributions presented in this study are included in the article/supplementary material, further inquiries can be directed to the corresponding author.

## Ethics statement

Ethical review and approval was not required for the study on human participants in accordance with the local legislation and institutional requirements. Written informed consent for participation was not required for this study in accordance with the national legislation and the institutional requirements.

## Author contributions

BZ: conceptualization. J-WZ, J-GG, W-ES, and Z-HP: data curation. J-WZ, Y-CG, and BZ: formal analysis. J-WZ, M-MW, and BZ: methodology. X-GS and BZ: project administration. YL, Y-ML, Q-QS, BL, and BZ: supervision. J-WZ, YL, W-ES, and C-YY: writing—original draft. YL, Y-ML, and BZ: writing—review and editing. All authors contributed to the article and approved the submitted version.

## Footnotes

1
http://www.cdc.gov/nchs/nhanes.htm
